# Energy Efficient Sensor Scheduling with a Mobile Sink Node for the Target Tracking Application

**DOI:** 10.3390/s90100696

**Published:** 2009-01-23

**Authors:** Suhinthan Maheswararajah, Saman Halgamuge, Malin Premaratne

**Affiliations:** 1 Department of Mechanical Engineering, Melbourne School of Engineering, University of Melbourne, Australia; E-mail: saman@unimelb.edu.au (S.H.); 2 Advanced Computing and Simulation Laboratory, Department of Electrical and Computer Systems Engineering, Monash University, Clayton, Victoria, Australia; E-mail: malin@eng.monash.edu.au (M.P.)

**Keywords:** Sensor Scheduling, Dynamic Programming, Markov Model, Target Tracking

## Abstract

Measurement losses adversely affect the performance of target tracking. The sensor network's life span depends on how efficiently the sensor nodes consume energy. In this paper, we focus on minimizing the total energy consumed by the sensor nodes whilst avoiding measurement losses. Since transmitting data over a long distance consumes a significant amount of energy, a mobile sink node collects the measurements and transmits them to the base station. We assume that the default transmission range of the activated sensor node is limited and it can be increased to maximum range only if the mobile sink node is out-side the default transmission range. Moreover, the active sensor node can be changed after a certain time period. The problem is to select an optimal sensor sequence which minimizes the total energy consumed by the sensor nodes. In this paper, we consider two different problems depend on the mobile sink node's path. First, we assume that the mobile sink node's position is known for the entire time horizon and use the dynamic programming technique to solve the problem. Second, the position of the sink node is varied over time according to a known Markov chain, and the problem is solved by stochastic dynamic programming. We also present sub-optimal methods to solve our problem. A numerical example is presented in order to discuss the proposed methods' performance

## 1. Introduction

In a sensor network, a large number of small, low cost sensing devices called sensor nodes are deployed and interconnected to gather information from the field of interest and transmit it to the base station. The gathered information may be used to perform various kinds of tasks, such as environmental monitoring, habit monitoring, intelligent building and military applications. Each sensor node in the sensor field is powered by a battery and consumes energy for various purposes such as sensing, on board signal processing and transmitting. Energy required for transmission is significantly more than other purposes and is a function of the distance between sender and receiver. In general, sensor nodes are stationary and unattended. It is not easy and in most cases impractical to replace or recharge the battery in hazardous environments [1, 2]. Once the battery runs out, the sensor node becomes inoperable, increasing the coverage hole and decreasing the connectivity of the whole network [2, 3], on which the sensor network's performance depends highly. Therefore, efficient use of the sensor node battery's energy is an important aspect of sensor networks.

The life span of a sensor network is determined by the time duration until the sensor network fails to function due to inadequate number of sensor nodes [2, 4]. Many strategies have been proposed to prolong the life span of sensor networks [5, 6, 7, 8]. Activating a set of sensor nodes and putting the rest of the sensor nodes in *sleep* mode is a technique widely used to prevent unwanted energy consumption. In [9], the low-energy adaptive clustering hierarchy (LEACH) was proposed to distribute the energy consumption evenly throughout the sensor nodes, doubling the life span of the network. The alternative strategies for long distance transmission, such as multi-hop transmission and using a mobile sink node to perform the transmission [10, 11] also save significant energy. Data latency is experienced in multi-hop transmission, and the lower the latency the better the performance, especially in time-critical applications. Generally a mobile sink node has more battery energy, but navigating the mobile sink node is a challenging task. Many solutions have been proposed to solve this navigation problem [12, 13].

In target tracking, continuous collection of information about the target improves the tracking quality. The loss of the target and the measurement losses seriously affect the tracking quality [14]. Moreover in [15], an information theoretic approach was proposed to estimate the number of targets and their states simultaneously, where the expected information from the targets are maximized by selecting appropriate sensor nodes. In this paper, we show that for a linear Gaussian dynamics, the measurement losses adversely affect the tracking quality. In literature, the sensor scheduling was mainly studied in two different ways: first, scheduling the sensor nodes to minimize the tracking error subject to limited sensor usage and communication costs. Second, scheduling the sensor nodes to minimize the cost of sensor usage and communication subject to constraints on tracking quality. Our previous work [16] focused on maximizing the tracking quality, subject to limited battery energy available at the sensor nodes and we used several techniques to solve this constrained problem. In [17], the energy constraint was relaxed using Lagrangian multipliers and the problem was solved using an approximate dynamic programming technique. Predicting the target and activating a set of sensor nodes closer to the target, rather than activating all the sensor nodes, conserves energy in the sensor network and energy efficient sensor scheduling algorithm was presented in [18]. The optimal sensor scheduling was considered as minimizing the usage of sensor resources (i.e., battery energy and number of sensor nodes) whilst keeping the tracking accuracy at a desired level [19, 20, 21].

In this study, we minimize the total energy consumed by the sensor nodes whilst avoiding the additional error in the estimation due to the measurement losses. A mobile sink node is used to collect the measurements and transmit them to the base station (BS). The BS schedules the sensor nodes to send the measurements to the mobile sink node without loss considering the minimal energy consumption. In many situations, multiple sensor nodes cannot be activated at the same time due to the limited bandwidth or avoid interferences between sensor nodes. For example sonar sensor nodes cannot be operated simultaneously in the same frequency band in order to avoid interferences [22] and, therefore, a single sensor node is allowed to activate at each time step. In [23], it was assumed that once a sensor node is activated it remains active for a certain time period and can only be changed to another sensor node if the active time period of a sensor node is elapsed. Moreover switching between different types of sensor nodes caused additional cost [24]. In this paper, we assume that a single sensor node can be activated at any time step and each sensor node has an active time period. Therefore, a sensor node cannot be activated during the active period of a previously activated sensor node. We use deterministic and stochastic dynamic programming to solve this problem optimally when the path of the mobile sink node is known fully and partially, respectively. Moreover, we propose sub-optimal methods for both cases, and discuss the performance of the proposed methods with respect to the end result as well as to the computational cost.

The remainder of this paper is organized as follows. Section II constitutes the overview of our problem and analyzes the effect of measurement losses on tracking quality for a linear Gaussian dynamics. In section III, the assumptions and constraints made for this study are presented to formulate the sensor management problem. We describe optimal and sub-optimal methods to solve the sensor management problem for fully and partially known mobile sink node's paths. In Section IV, a numerical example of our problem is presented to analyze the performance and limitations of the proposed methods. Finally, our conclusion and future work are presented in section V.

## 2. Problem description and Models

**Figure 1.**
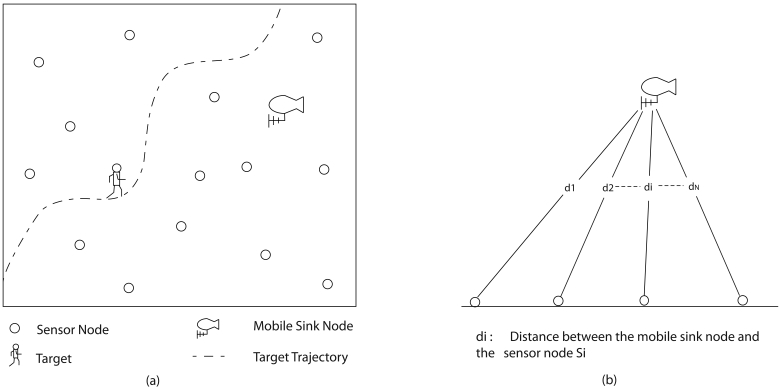
(a) Tracking a single target using multiple sensor nodes and a mobile sink node in the sensor network. (b) The Euclidean distances between the sensor nodes and the mobile sink node.

Figure 1 shows a wireless sensor network which is located far form the base station. Several sensors are deployed to track a target which moves in the field. In each time step a known set of sensor nodes cover the target and the sensor measurements are transmitted to a mobile sink node, for example an unmanned aerial vehicle (UAV) is flying over the sensor field. The mobile sink node transmits the collected measurements directly to the base station. The energy required for a sensor node to transmit the measurement depends on the distance between the sensor node and the mobile sink node. The distance is illustrated in Figure 1(b). An activated sensor node far away from the mobile sink node consumes more energy to transmit the measurement, compared to one closer to the mobile sink node and therefore the energy is wasted. Alternatively we may follow two approaches when the activated sensor node is far away from the mobile sink node: first, activating another sensor node closer to the mobile sink node: Second, not transmitting the measurement over a long distance. Once a sensor node is activated it cannot be changed to another one for a certain time period and therefore the first approach is void. Since measurement losses degrade the tracking quality, the second approach is strictly avoided. Therefore, the base station should manage the sensor nodes to minimize the total waste of energy without degrading the tracking quality. The energy model of the sensor node and the importance of availability of measurements for target tracking are presented below.

### 2.1. Sensor Model

The base station (BS) schedules the sensor nodes to either *sleep* or *active* mode to perform the tasks efficiently. In order to satisfy the limited bandwidth, one sensor node (or generally a set of sensor nodes) is in *active* mode at any given instant. We assume that the position of the target belongs to a region denoted by Ω*_k_* at time step *k* and it is known a priori. Let {S1, S_2_, …, S_N_*_k_*} denote the set of feasible sensor nodes for scheduling at time step *k* and sensor node S*_i_* is an element in the set {S_1_, S_2_, …, S_N_} only if its sensing range covers the region Ω*_k_*. Here, N*_k_* is the total number of feasible sensor nodes available for the scheduling at time step *k*. If a sensor node S*_i_* is activated at time step *k*, the BS cannot activate another sensor node S*_j_* for a period of *t_i_* time steps (ie, BS can only activate the S*_j_* sensor node at *k* + *t_i_* time step or later). Each sensor node consumes energy for sensing, signal processing and transmitting. The energy consumption of the sensor node during its *active* and *sleep* modes is assumed as follows:

(1) $$\psi =\{\begin{array}{ccc} \psi_{i+\psi_{t}} & \text{if} & \text{active}\\ \psi_{2} & \text{if} & \text{sleep} \end{array}$$

The power consumption by transmission is denoted by *ψ_t_* and *ψ*_1_ represents the power required for sensing and signal processing. The power consumption due to its own timer during the *sleep* mode is denoted by *ψ*_2_. In *active* mode the sensor node consumes more energy than in *sleep* mode, due to particularly the transmission. In order to transmit *r* bits of data to a distance of *d*, the sensor node requires (*α*_1_ + *α*_2_*d*^2^)*r* energy at each time step [25], where *α*_1_ denotes the electronic energy required to transmit one bit of data and *α*_2_ is a constant related to the radio energy The transmission range of the sensor node S*_i_* is set at default range *r_i_*. However, it can be set at $r_{i}^{\max}(>r_{i})$ only when the mobile sink node is not reachable by the sensor node with *r_i_*. The intuition behind this method is to prolong the life span of the sensor network by avoiding unnecessary long distance transmissions.

Moreover, the sensor nodes' measurements are linearly related to the state of the target and corrupted by white Gaussian noise. The measurement from the sensor node S*_i_* at time step *k* is given by:

(2) $${\text{y}}_{k}^{i}={\text{H}}^{i}{\text{X}}_{k}+\upsilon_{k}^{i}$$

The column vector X*_k_* = [*χ_k_ χ̇_k_ ξ_k_ ξ̇_k_*]′ denotes the state of the target at time step *k* where *χ_k_* and *ξ_k_* represent its positions in the *x* and *y* directions, *χ̇_k_* and *ξ̇_k_* represent its velocities in the *x* and *y* directions. The observation matrix is denoted as H*^i^*. The measurement noise $\upsilon_{k}^{i}$ for each sensor node is assumed to be independent of each other and $\upsilon_{k}^{i}∼N(0,R_{i})$. Here R*_i_* denotes the error covariance of the sensor node S*_i_*. In this study, we use homogeneous sensor nodes and therefore, the error covariance R*_i_* and the observation matrix H*^i^* are the same for all the sensor nodes and are known a priori.

### 2.2. Tracking Quality and Measurement availability

In this sub-section, we analyze the consequences of measurement losses in target tracking applications. Let X*_k_* represent the state of the target at time step *k*. Then the motion of the target can be modeled by a stochastic equation. In many applications, the stochastic equation is assumed linear [16, 26], non linear [27] and Markov process [28, 29]. In this study, we assume that the target evolves according to the linear stochastic equation impaired by white Gaussian noise and given by:

(3) $${\text{X}}_{k+1}={\text{FX}}_{k}+w_{k}$$

where *w_k_* is the white Gaussian process noise with a known covariance Q and the intensity of the process noise is determined by the scalar quantity *q*. F denotes the system matrix which models the state kinematics of the target. For a nearly constant velocity model of the target, F and Q are given by [30]:

$$F=\left[\begin{array}{cccc} 1 & \Delta t & 0 & 0\\ 0 & 1 & 0 & 0\\ 0 & 0 & 1 & \Delta t\\ 0 & 0 & 0 & 1 \end{array}\right],\;Q=q\left[\begin{array}{cccc} \frac{\Delta t^{3}}{3} & \frac{\Delta t^{2}}{2} & 0 & 0\\ \frac{\Delta t^{2}}{2} & \Delta t & 0 & 0\\ 0 & 0 & \frac{\Delta t^{3}}{3} & \frac{\Delta t^{2}}{2}\\ 0 & 0 & \frac{\Delta t^{2}}{2} & \Delta t \end{array}\right].$$

The state of the target evolves after Δ*t* time steps. Since the state of the target and the measurements are assumed to have linear Gaussian dynamics, the Kalman filter is used to optimally estimate the state and calculate its error covariance [31]. If the initial state of the target X_0_ is known with the error covariance P_0_, the estimated state of the target *ξ_k∣k_* = 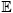 {X*_k_*} and its error covariance P*_k∣k_* = 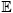 {(X*_k_* − *ξ_k∣k_*) (X*_k_* − *ξ_k∣_k*)′} at time step *k* are given by:

(4) $$\xi_{k|k}=\xi_{k|k-1}+{\text{K}}_{k}(y_{k}-z_{k|k-1})$$

(5) $${\text{P}}_{k|k}=(I-{\text{K}}_{k}\text{H}){\text{P}}_{k|k-1}$$

where

*ξ_k_*_∣_*_k_*_−1_ = F*ξ_k_*_−1∣_*_k_*_−1_

*z_k_*_∣_*_k_*_−1_ = H*ξ_k_*_−1∣_*_k_*_−1_.

The predicted state and the predicted measurement at time step *k* − 1 are denoted as *ξ_k_*_∣_*_k_*_−1_ and *z_k_*_∣_*_k_*_−1_ respectively. The predicted error of the estimated state P*_k_*_∣_*_k_*_−1_ and the Kalman gain K*_k_* at time steps *k* − 1 and *k* are given as:

(6) $${\text{P}}_{k|k-1}={\text{FP}}_{k-1|k-1}F^{\prime }+\text{Q},$$

(7) $${\text{K}}_{k}={\text{P}}_{k|k-1}H^{\prime }{\left(\text{R}+{\text{HP}}_{k|k-1}H^{\prime }\right)}^{-1}.$$

It can be inferred from (5) and (7) that the error covariance of the estimated state is influenced by the error covariance R of the sensor node. In [16], we studied the problem of sensor management to enhance the tracking quality, and showed that the tracking quality depends on the sequence of the activated sensor nodes where the error covariances of the sensor nodes are different. However, the tracking quality does not depend on the sequence of activated sensor nodes if the error covariances of the sensor nodes are the same and the measurements are available at each time step.

In state estimation, measurements may be absent for many reasons, such as that the target is not covered by any sensor nodes or the sensed measurement is lost. Considering the limited transmission range of each sensor node, it is possible that measurements may not be collected by the mobile sink node when it is outside the transmission range of the activated sensor node. Let E*_k_* and ${\text{E}}_{k}^{\text{lost}}$ denote the RMSE (root mean square error) of the estimated state at time step *k* without and with measurement loss, respectively. Thus E*_k_* is the square root of the estimated error for availability of continuous measurements and ${\text{E}}_{k}^{\text{lost}}$ is the square root of estimation error for availability of noncontinuous measurements. We can calculate E*_k_* and ${\text{E}}_{k}^{\text{lost}}$ as follows:

(8) $${\text{E}}_{k}=\sqrt{{\text{P}}_{k|k}}$$

(9) $${\text{E}}_{k}^{\text{lost}}=\{\begin{array}{lll} \sqrt{{\text{P}}_{k|k}} & \text{if} & \text{measurement is received}\\ \sqrt{{\text{P}}_{k|k-1}} & \text{if} & \text{measurement is lost}. \end{array}$$

**Figure 2.**
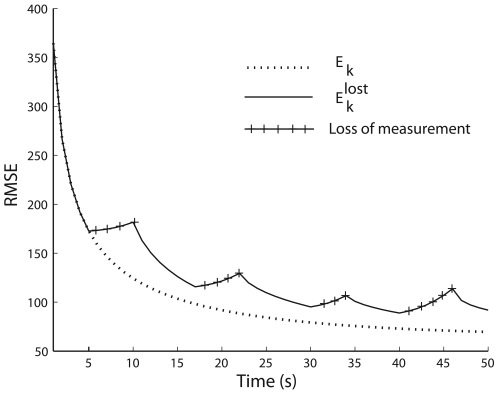
Variation of the RMSE of the estimated state with and without measurement loss.

For observable [F, H] and controllable [F, Q^½^] the RMSE of the estimated state E*_k_* asymptotically reaches a steady state if there is no measurement loss. The variation of E*_k_* and ${\text{E}}_{k}^{\text{lost}}$ with time are illustrated in Figure 2, and it can be seen that E*_k_* reaches to a steady state error, but not ${\text{E}}_{k}^{\text{lost}}$. Here, we used Δ*t* = 1s, *q* = 10, observation matrix as 4 × 4 identity matrix and the error covariance of the sensor nodes as:

$$\text{R}=\text{diag}\;\left[\begin{array}{c} 2.25\times 10^{3}\\ 100\\ 1.5\times 10^{5}\\ 100 \end{array}\right]$$

Since P*_k∣k_*_−1_ ≥ P*_k∣k_*, we can say that ${\text{E}}_{k}^{\text{lost}}\geq {\text{E}}_{k}$ at any time step *k* as it can be seen in Figure 2. The additional ${\text{E}}_{k}^{\text{lost}}-{\text{E}}_{k}$ error in estimation is due to the measurement loss. Moreover, we consider three different cases where the measurements are lost at different time steps and the number of measurement losses are not equal. The measurement losses occurred at *k* = 5, 6, 7, 8, *k* = 14, 16, 18, 20, 22 and *k* = 84, 86, 88, 90, 92 for case 1, case 2 and case 3 respectively. We denote the RMSE of the estimated error as ${\text{E}}_{k}^{{\text{lost}}_{1}}$, ${\text{E}}_{k}^{{\text{lost}}_{2}}$ and ${\text{E}}_{k}^{{\text{lost}}_{3}}$ for case 1, case 2 and case 3 respectively.

**Figure 3.**
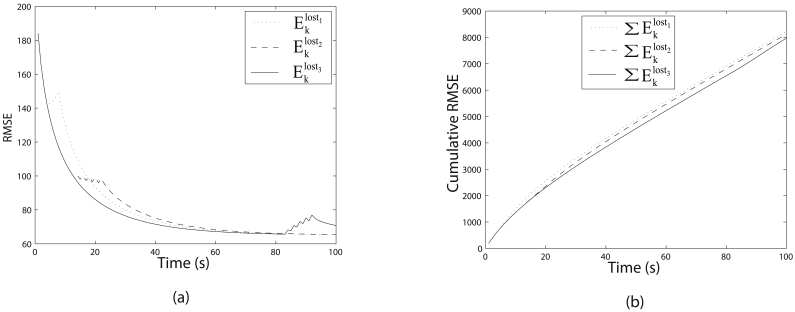
(a) Variation of the RMSE of the estimated state with measurement losses happening at different times. (b) Variation of cumulative RMSE of the estimated state.

The variation in RMSE and cumulative RMSE are illustrated in Figure 3. Even though the number of measurement losses are equal for case 2 and case 3, the cumulative RMSE are not the same. Furthermore, the number of measurement losses for case 1 is less than that of case 2 and case 3, but produces a higher cumulative RMSE than case 2 and case 3. This indicates that the time steps of measurement losses are more important than the number of measurement losses for target tracking. Moreover, it can be seen in Figures 2 and 3 that measurement losses creates unnecessary error in estimation resulting in degraded tracking quality. Therefore, we make sure that the measurements are not lost at any time step to avoid the additional error in estimation. We increase the transmission range of the sensor nodes to avoid measurement loss only if necessary as this has to be done with minimal energy consumption of the sensor nodes. The following section describes the sensor management strategies according to the mobile sink node positions.

## 3. Sensor Management with Mobile Sink Node

Let L*_i_* = (*l_xi_, l_yi_, l_zi_*) and M*_k_* = (*m_xk_, m_yk_, m_zk_*) denote the position of the sensor node S*_i_* and the mobile sink node in 3-dimensional Cartesian space at time step *k* respectively. The assumptions and constraints made in this study are as follows:

1. The position of the target belongs to a known region Ω*_k_* at time step *k* and the sensor set {S_1_, S_2_, …, S_N_*_k_*} which covers the region Ω*_k_* is known a priori
2. Only a single sensor node can be *active* mode at any time step.
3. A sensor node can not be activated during the *active* period *t_i_* of the previously activated sensor node S*_i_*.
4. The transmission range of the activated sensor node S*_i_* is set at $r_{i}^{\max}$ only if ‖L*_i_* − M*_k_*‖ *> r_i_* otherwise it remains at default range *r_i_*.
5. $r_{i}^{\max}$ is selected in such a way that the mobile sink node is always within the range. Thus, ‖L*_i_* − M*_k_*‖ < $r_{i}^{\max}\forall {\text{S}}_{i}$ and ∀ *k*.

Our objective is to minimize the total energy consumed by the sensor nodes in order to send the measurements to the mobile sink node without loss. We define the cost function *J*_T_ for the entire time horizon {1, 2, .., T} as follows:

(10) $$J_{\text{T}}=\sum_{k=1}^{\text{T}}\left\{\delta_{u_{k}}^{{\text{M}}_{k}}\left(\psi_{u_{k}}^{\max}-\psi_{u_{k}}\right)+\psi_{u_{k}}\right\}$$

where *u_k_* denotes the sensor node in *active* mode at time step *k* and *u_k_* ∈ {S_1_, S_2_, …, S_N_*_k_*}. The measurement-loss function with the default transmission range is denoted as $\delta_{u_{k}}^{{\text{M}}_{k}}$ and given by:

$$\delta_{u_{\text{k}}}^{{\text{M}}_{\text{k}}}=\{\begin{array}{lll} 0 & \text{if} & \left\|{\text{L}}_{u_{k}}-{\text{M}}_{k}\right\|\leq r_{u_{k}}\\ 1 & \text{if} & \left\|{\text{L}}_{u_{k}}-{\text{M}}_{k}\right\|>r_{u_{k}}. \end{array}$$

This problem would be easily solved if the mobile sink node were always reachable by a single sensor node with a lower transmission range than the rest of the sensor nodes. However, it may be impossible to find such a sensor node in practical terms. Our aim is to find the optimal sequence of activated sensor nodes $\left\{u_{1}^{*},u_{1}^{*},\ldots u_{T}^{*}\right\}$ to minimize the total accumulated cost *J*_T_ from time 1 to T:

(11) $$\left\{u_{1}^{*},u_{1}^{*},\ldots u_{\text{T}}^{*}\right\}=\arg\underset{\forall u_{k}}{\min}\sum_{k=1}^{\text{T}}\left\{\delta_{u_{k}}^{{\text{M}}_{k}}\left(\psi_{u_{k}}^{\max}-\psi_{u_{k}}\right)+\psi_{u_{k}}\right\}.$$

Since *J*_T_ is a function of the relative position of the mobile sink node, we solve this optimization problem for cases when the mobile sink node's position is known both fully and partially.

### 3.1. Position of the mobile sink node is known

Let {M_1_, M_2_, …, M_T_} denote the sequence of the position of the mobile sink node. We assume that it is known a priori. The base station (BS) collects information about the sensor nodes, such as location and the energy availability. We solve this problem using a dynamic programming technique and rollout algorithm as follows.

#### a. Dynamic Programming Approach

We used deterministic backward dynamic programming (DP) to find the optimal sequence of the activated sensor nodes. DP is a recursive technique which divides the problem into a sequence of sub-problems using principle of optimality. In this paper, *state* refers the position of the mobile sink node, *stage* refers the time step and *decision* refers the activated sensor node. Since the *state* M*_k_* is known at the *k*th *stage*, this problem has only a single *state* at each *stage*. The *decision* at time step *k* when the position of the mobile sink node is at M*_k_* is denoted as *μ_k_*(M*_k_*) and the number of *decisions* are equal to the number of available sensor nodes.

If the *decision* at time step *k* is made to activate *μ_k_*(M*_k_*) = *u_k_* (i.e, to activate the sensor node *u_k_* at time step *k*), then the period cost (please note that it is not the instantaneous cost but the period cost because once the sensor node has been activated it remains in *active* mode for *t_i_* time steps) from *k* to *k* + *t_i_* − 1 is given by:

(12) $$g_{k}(u_{k})=\sum_{t=k}^{\min(k+t_{u_{k}}-1,\text{T})}\left\{\delta_{u_{k}}^{{\text{M}}_{k}}\left(\psi_{u_{k}}^{\max}-\psi_{u_{k}}\right)+\psi_{u_{k}}\right\}.$$

We can rewrite our objective function *J*_T_ as:

(13) $$J_{\text{T}}=\sum g_{k}(\mu_{k}({\text{M}}_{k}))\;k=1:t_{\mu_{k}({\text{M}}_{k})}:\text{T}.$$

The DP proceeds backwards from *stage* T to *stage* 1 and the “cost-to-go” functions are defined as follows:

(14) $$J_{\text{T}}({\text{M}}_{\text{T}})=\underset{\forall \mu_{\text{T}}({\text{M}}_{\text{T}})}{\min}\left\{\delta_{\mu_{\text{T}}({\text{M}}_{\text{T}})}^{{\text{M}}_{\text{T}}}(\psi_{\mu_{\text{T}}({\text{M}}_{\text{T}})}^{\max}-\psi_{\mu_{\text{T}}({\text{M}}_{\text{T}})})+\psi_{\mu_{\text{T}}({\text{M}}_{\text{T}})}\right\}\;\text{at}\;\text{stage}\;\text{T}$$

(15) $$J_{k}({\text{M}}_{k})=\underset{\forall \mu_{k}({\text{M}}_{k})}{\min}\left\{g_{k}(\mu_{k}({\text{M}}_{k}))+J_{k+\text{K}}({\text{M}}_{k+\text{K}})\right\}\;\text{at}\;\text{stage}\;\text{T}-1,\ldots 2,1$$

where

K = *t_μk_*_(M_*_k_*_)_.

DP solves the sub-problems from *k* = T to 1 and stores the optimal value for *J_k_*(*M_k_*) and $\mu_{k}^{*}({\text{M}}_{\text{k}})$. Once all the sub-problems have been solved, DP backtracks the optimal sensor nodes from the stored values. The optimal total cost is given by:

(16) $$\min J_{\text{T}}=J_{1}({\text{M}}_{1}).$$

The optimal sensor node to be activated at time step 1 and *k* are given by:

(17) $$u_{1}^{*}=\arg\{J_{1}({\text{M}}_{1})\}$$

(18) $$u_{k}^{*}=\{\begin{array}{lll} {\text{S}}_{j} & \text{if} & {\text{S}}_{j}\;\text{sensor node is in}\;\text{active}\;\text{mode}\\ \arg\{J_{k}({\text{M}}_{k})\} & \text{if} & \text{otherwise} \end{array}$$

where S*_j_* denotes the previously activated optimal sensor node. For this problem, the computational cost of DP only depends on the number of *stages* (total time horizon) and the number of admissible *decisions* (available feasible sensor nodes). Since the DP calculations are done off-line, it is assumed that at each time step set of available feasible sensor nodes are known a priori. In real situations, the available feasible number of sensor nodes can vary with time, for example sensor nodes may be deleted due to dead battery or malfunction. In this study, we assume that the set of available feasible sensor nodes at each time step are known and the sensor nodes work without malfunction.

#### b. Rollout Approach

The rollout algorithm (RA) is an approximate dynamic programming technique to overcome the curse of dimensionality in DP. The algorithm performs according to the approximate cost-to-go function given by a sub-optimal base heuristic policy. It produces a solution not worse than the solution given by the base heuristic. The computational cost of the RA depends on the problem size and the base heuristic method. In general, RA is computationally more tractable than DP. Since M*_k_* has a single value at each time step, known in our problem, the number of *states* at each *stage* is one and therefore, the computational cost of DP is not an issue. However, DP may not be used if the *decision* variables are varying with time. RA updates *stages, states* and *decisions* at each time step and performs the optimization. The major steps involved in RA for our problem are as follows:

**Step1:** At *k* = 1, update {S_1_, S_2_, …, S_N_1__} and calculate *g*_1_(S*_i_*) + *J*_1+_*_t_i__*(M_1+_*_t_i__*), ∀ S*_i_* ∈ {S_1_, S_2_, …, S_N_1__}. Here, *J*_1+_*_t_i__*(M_1+_*_t_i__*) is solved by the base heuristic method.
**Step 2:** Choose the sensor node ${\tilde{u}}_{1}^{*}$ which minimizes *g*_1_(*i*) + *J*_1+_*_t_i__*(M_1+_*_t_i__*).
**Step 3:** At time *k*, update {S_1_, S_2_, …, S_N_}.
**Step 4:** If a sensor node S*_j_* is in *active* mode set ${\tilde{u}}_{k}^{*}={\text{S}}_{j}$ and go to Step 3 with replace *k* by *k* + 1, else go to Step 5.
**Step 5:** Calculate *g_k_*(S*_i_*) + *J_k_*_+_*_t_i__* (M*_k_*_+_*_t_i__*) for all ∀ S*_i_* ∈ {1, 2, .., N*_k_*} and *J_k_*_+_*_t_i__*(M*_k_*_+_*_t_i__*) is solved by the base heuristic method.
**Step 6:** choose the sensor node ${\tilde{u}}_{k}^{*}$ which minimizes *g_k_*(S*_i_*) + *J_k_*_+_*_t_i__*(M*_k_*_+_*_t_i__*).
**Step 7:** If *k* > T go to Step 8, else go to Step 3, replacing *k* with *k* + 1.
**Step 8:** The sub-optimal sensor node sequence is given by $\left\{{\tilde{u}}_{1}^{*},{\tilde{u}}_{2}^{*},\ldots ,{\tilde{u}}_{\text{T}}^{*}\right\}$.

We use the one-step-look ahead (OSLA) [32] method as our base heuristic method for RA. Since OSLA algorithm optimizes only the cost occurred at the current time step, the results are sub-optimal and the computational cost required is comparatively very small. The OSLA algorithm produces the sub-optimal sensor sequence $\{{\overset{⌣}{u}}_{1}^{*},{\overset{⌣}{u}}_{2}^{*},\ldots ,{\overset{⌣}{u}}_{\text{T}}^{*}\{$ and given by:

(19) $${\overset{⌣}{u}}_{1}^{*}=\arg\underset{\forall \mu_{1}({\text{M}}_{1})}{\min}\left\{\delta_{\mu_{1}({\text{M}}_{1})}^{{\text{M}}_{1}}(\psi_{\mu_{1}({\text{M}}_{1})}^{\max}-\psi_{\mu_{1}({\text{M}}_{1})})+\psi_{\mu_{1}({\text{M}}_{1})}\right\}$$

(20) $${\overset{⌣}{u}}_{k}^{*}=\{\begin{array}{lll} {\text{S}}_{j} & \text{if} & {\text{S}}_{j}\;\text{sensor node is in}\;\text{active}\;\text{mode}\\ \arg\underset{\forall \mu_{k}({\text{M}}_{k})}{\min}\left\{\delta_{\mu_{k}({\text{M}}_{k})}^{{\text{M}}_{k}}(\psi_{\mu_{k}({\text{M}}_{k})}^{\max}-\psi_{\mu_{k}({\text{M}}_{k})})+\psi_{\mu_{k}({\text{M}}_{k})}\right\} & \text{if} & \text{otherwise} \end{array}$$

where S*_j_* denotes the previously activated optimal sensor node.

### 3.2. Position of the mobile sink node is partially known

In this section, we assume that the exact position of the mobile sink node M*_k_* is known only at time step *k* otherwise it is only partially known. In other words, M*_k_* is known ∀*k* ≤ *c* and partially known ∀*k* > *c* at current time step *c*. We assume that the BS immediately gets to know the current position of the mobile sink node at time step *k*.

The position of the mobile sink node varies with time according to a known Markov chain. Assume that M*_k_* is an *S*-state Markov chain with the state space {*e*_1_, …, *e_S_*}. Here, *e_s_* denotes a position in 3-dimensional Cartesian space. The transition probability matrix A and the initial probability vector *π*_1_ of the mobile sink node's position are defined as:

A = [*a_mn_*]*_S×S_* where *a_mn_* = *P*(M*_k_* = *e_n_∣*M*_k_*_−1_ = *e_m_*), *m, n* ∈ {1, …,*S*}.

*π*_1_ = [*π*_1_(*m*)]*_S×_*_1_ where *π*_1_(*m*) = *P*(M_1_ = *e_m_*), *m* ∈ {1, …,*S*}.

The Markov chain parameters *A* and *π*_1_ are assumed to be known.

#### a. Stochastic Dynamic Programming Approach

Since the exact future position of the mobile sink node is unknown at the current time step, we cannot use the deterministic dynamic programming for this problem. We present the stochastic dynamic programming (SDP) technique to produce the optimal sequence of activated sensor nodes when the mobile sink node' future position is uncertain. Let *g_k_*(S*_i_*, M*_k_*) denotes the expected period cost from *k* to *k* + *t_i_* − 1 when the position of the mobile sink node is in M*_k_* and the activated sensor node at *k*th time step is S*_i_*. The period cost *g_k_*(S*_i_*, M*_k_*) is given by:

(21) $$g_{k}({\text{S}}_{i},{\text{M}}_{k})=\sum_{t=k}^{\min(k+t_{i}-1,\text{T})}\underset{t\neq k}{E}\left\{\delta_{i}^{{\text{M}}_{t}}(\psi_{i}^{\max}-\psi_{i})+\psi_{i}\right\}.$$

The future position of mobile sink node M*_t_* is unknown ∀*t* > *k* at time step *k* and therefore, we take the expectation to calculate the period cost *g_k_*(S*_i_*, M*_k_*).

**Figure 4.**
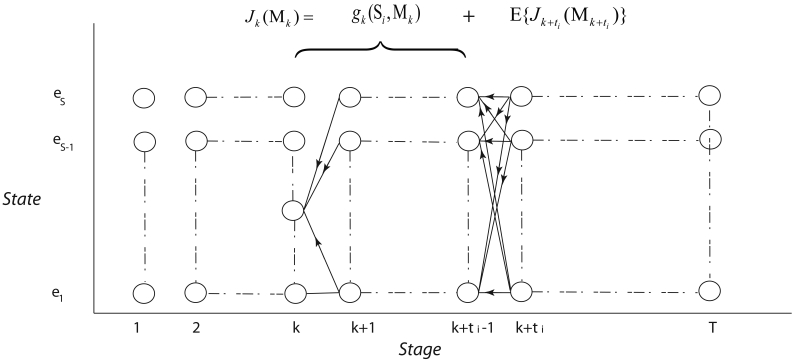
*state-stage* diagram of SDP with S number of *states* and T number of *stages*.

For this problem M*_t_* can take any state in *S*-state Markov chain and therefore, SDP has *S* number of *states* at each *stage*. Figure 4 shows the *state – stage* diagram of the SDP for this sensor management problem. The SDP proceeds backwards from *stage* T to *stage* 1 and the “cost-to-go” functions are defined as follows:

(22) $$J_{\text{T}}({\text{M}}_{\text{T}})=\underset{\forall \mu_{\text{T}}({\text{M}}_{\text{T}})}{\min}\left\{\delta_{\mu_{\text{T}}({\text{M}}_{\text{T}})}^{{\text{M}}_{\text{T}}}(\psi_{\mu_{\text{T}}({\text{M}}_{\text{T}})}^{\max}-\psi_{\mu_{\text{T}}({\text{M}}_{\text{T}})})+\psi_{\mu_{\text{T}}({\text{M}}_{\text{T}})}\right\}\;\text{at}\;\text{stage}\;\text{T}$$

(23) $$J_{k}({\text{M}}_{k})=\underset{\underset{\forall {\text{M}}_{k+\text{K}}}{\forall \mu_{k}({\text{M}}_{k})}}{\min}\left\{g_{k}(\mu_{k}({\text{M}}_{k}),{\text{M}}_{k})+E\left\{J_{k+\text{K}}({\text{M}}_{k+\text{K}})\right\}\right\}\;\text{at}\;\text{stage}\;\text{T}-1,\ldots 2,1$$

where

K = *t*_*μ_k_*(M_*k*_)_.

SDP solves the sub-problems from *k* = T to 1 and stores the values for *J_k_*(M*_k_*) and $\mu_{k}^{*}({\text{M}}_{k})$. The optimal expected total cost *J*_T_ is given by:

(24) $$\min E\{J_{\text{t}}\}=\sum_{a=1}^{S}\pi_{1}(e_{a})J_{1}(e_{a}).$$

The optimal sensor node to be activated at time steps 1 and *k* depend on the position of the mobile sink node and is given by:

(25) $$\mu_{1}^{*}({\text{M}}_{1})=\arg\{J_{1}({\text{M}}_{1})\}$$

(26) $$\mu_{k}^{*}({\text{M}}_{k})=\{\begin{array}{lll} j & \text{if} & j\;\text{th sensor node is in}\;\text{active}\;\text{mode}\\ \arg\{J_{k}({\text{M}}_{k})\} & \text{if} & \text{otherwise} \end{array}$$

where *j* denotes the previously activated optimal sensor node. These calculations are done at the BS off-line. At each time step, the BS updates the current position of the mobile sink node and activates the optimal sensor node using the look up table, where the SDP stores *J_k_*(M*_k_*) and $\mu_{k}^{*}({\text{M}}_{k})$. The computational cost of SDP for this problem depends on the number of *stages*, number of *states* and number of admissible *decisions*. Furthermore, we assume that the number of available sensor nodes (admissible *decisions*) at each time step are known a priori and the sensor nodes work without malfunction.

#### b. One-step-look-ahead method

For a large problem (where the number of *states, stages* and *decisions* are larger), SDP may not solve the problem with-in an acceptable time period, due to its huge computational cost. Consider a scenario where the parameters required for the SDP are known just before the application (target started moving) and the problem is large enough that we may not have enough time to solve it before the application. Moreover, if the set of available sensor nodes varies with time, SDP cannot be used. We use the one-step-look-ahead (OSLA) algorithm as a sub-optimal method to solve this scheduling problem. The OSLA finds the best sensor node which minimizes only the energy consumption at the current time step for a known M*_k_* since M*_k_* is known at the current time step *k*. For *k* = 1, 2, … T, the OSLA produces the sub-optimal sensor sequence as $\left\{{\tilde{u}}_{1}^{*},{\tilde{u}}_{2}^{*},\ldots ,{\tilde{u}}_{\text{T}}^{*}\right\}$ where the sub-optimal sensor node at time step 1 and *k* are given by:

(27) $${\tilde{u}}_{1}^{*}=\arg\underset{\forall u_{1}}{\min}\left\{\delta_{u_{1}}^{{\text{M}}_{1}}(\psi_{u_{1}}^{\max}-\psi_{u_{1}})+\psi_{u_{1}}\right\}$$

(28) $${\tilde{u}}_{k}^{*}=\{\begin{array}{lll} j & \text{if} & j\;\text{th sensor node is in}\;\text{active}\;\text{mode}\\ \arg\underset{\forall u_{k}}{\min}\left\{\delta_{u_{k}}^{{\text{M}}_{k}}(\psi_{u_{k}}^{\max}-\psi_{u_{k}})+\psi_{u_{k}}\right\} & \text{if} & \text{otherwise} \end{array}$$

Although OSLA algorithm produces a sub-optimal solution for the sensor management problem, it is suitable when the availability of the feasible sensor nodes are unknown a priori or when we need to solve the problem within a short time period.

## 4. Results and Discussions

In this section, we present a numerical example of single target tracking with noisy sensor nodes and the BS is located far away from the sensor field. A mobile sink node flies around the sensor field, collects the measurements from the sensor nodes and transmits them to the BS in order to prolong the life span of the sensor network. We simulate a single target moving in a 2-dimensional Cartesian space according to (3). The system matrix F and the system noise covariance Q are the same as in section 2.2.

### 4.1. Parameter Settings for the Sensor Network

In our simulation, we use only 3 sensor nodes to enhance the clarity and simplicity of the problem. Sensor nodes S_1_, S_2_ and S_3_ are deployed in a sensor field of 500 m × 500 m and for the experimental purpose, we assume that each sensor node covers the target such that the feasible sensor set has S_1_, S_2_ and S_3_ sensor nodes at each time step. A mobile sink node collects the measurements and immediately transmits to the BS. The properties of the sensor nodes are given in Table 1.

It is assumed that the measurements are sent to the mobile sink node in a single-hop communication without any time delay. The observation matrix H is a unit matrix, and therefore the measurements are linearly related to the state of the target. The time interval between the measurements is considered as Δ*t* = 1s. We assumed the measurement data size as 1 MB and the data rate as 8 Mbps. Without loss of generality, we neglect the energy consumption *ψ*_1_ and *ψ*_2_ and only consider *ψ_tx_*. The parameters in *ψ_tx_* are set *α*_1_ = 50 nJ/b and *α*_2_ = 100 pJ/bm^2^. The maximum transmission range of $r_{i}^{\max}=500\text{m}$ was chosen for all sensor nodes. The target starts at X_0_ with the known initial error covariance P*_o_* and moves for T s.

### 4.2. Sensor management with known mobile sink node's path

**Figure 5.**
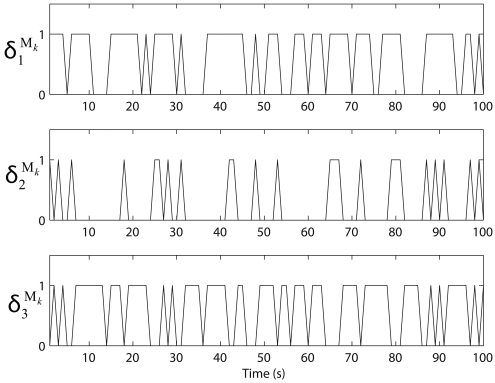
Measurement-loss functions with default transmission ranges of the sensor nodes with time.

In this section, we assume that the position of the mobile sink node is known at each time step and the mobile sink node flies horizontally at a height of 100 m above the ground. We plot the measurement-loss function $\delta_{i}^{{\text{M}}_{k}}$ with the *r_i_* for the total time horizon of 100s, shown in Figure 5.

**Figure 6.**
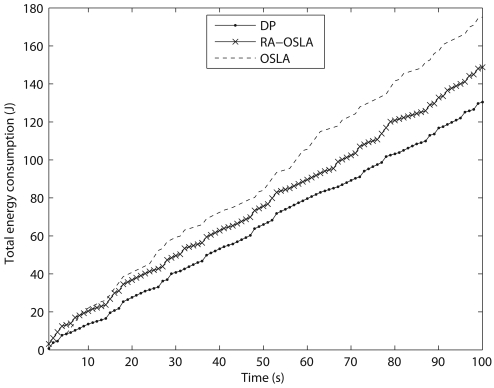
Variation of total energy consumption with time obtained by OSLA, RA-OSLA and DP.

We compare the sub-optimal solutions obtained by the rollout algorithm (RA) and one-step-look-ahead (OSLA) method with the optimal solution obtained by dynamic programming (DP). We use OSLA method as the base heuristic method for RA. We increase the total time horizon from T = 30s to 100s. The results for all three methods are shown in Table 2. The variation of total energy consumption with time is shown in Figure 6 for T = 100s. We can see from the results in Table 2 that RA always produces better results than the base heuristic method (OSLA). Since the positions of the mobile sink node are known a priori, the computational cost of DP is not large and therefore DP is best suited for this particular problem.

### 4.3. Sensor management with partially known mobile sink node's path

**Figure 7.**
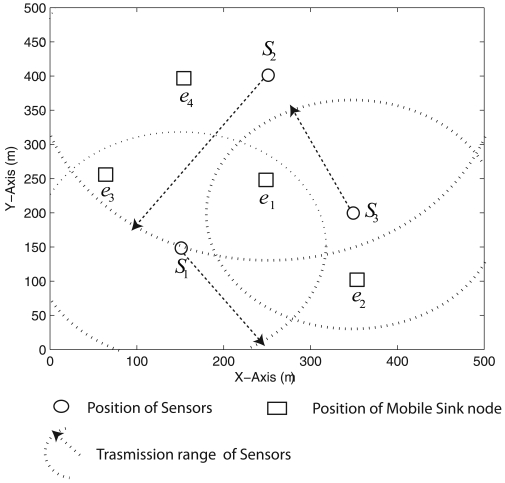
Probable positions of the mobile sink and the default transmission ranges and positions of the sensor nodes.

In this section, we assume that the position of the mobile sink node varies according to a known Markov chain and that the exact position of the mobile sink node is known only at the current time step but not before. We have chosen a 4-State Markov chain with the state space {*e*_1_, *e*_2_, *e*_3_, *e*_4_} for the position of the mobile sink node. Here *e*_1_ = (250, 250, 100)m, *e*_2_ = (350, 100, 100)m, *e*_3_ = (75, 250, 100)m and *e*_4_ = (150, 400, 100)m. Figure 7 shows the probable positions of the mobile sink node and of the sensor nodes, and the default transmission ranges of the sensor nodes

The initial probability vector of the mobile sink node's position is assumed as *π*_1_ = [0.2 0.4 0.2 0.2]. We choose two different transition probabilities to analyze the performance of our methods as follows:

$$A_{1}=\left[\begin{array}{cccc} 0.2 & 0.3 & 0.2 & 0.3\\ 0.3 & 0.3 & 0.2 & 0.2\\ 0.2 & 0.3 & 0.2 & 0.3\\ 0.3 & 0.1 & 0.2 & 0.4 \end{array}\right],\;A_{2}=\left[\begin{array}{cccc} 0.1 & 0.5 & 0.1 & 0.3\\ 0.1 & 0.3 & 0.1 & 0.5\\ 0.3 & 0.1 & 0.5 & 0.1\\ 0.1 & 0.3 & 0.1 & 0.5 \end{array}\right]$$

The optimal expected total energy consumption of the sensor nodes with A_1_ and A_2_ for T = 100s are calculated off-line using stochastic dynamic programming (SDP), and are given by:

$$E\left\{{\text{J}}_{100}^{{\text{A}}_{1}}\right\}=\sum_{a=1}^{4}\pi_{1}(a)J_{1}(e_{a})=142.54\text{J},\;E\left\{J_{100}^{{\text{A}}_{2}}\right\}=\sum_{a=1}^{4}\pi_{1}(a)J_{1}(e_{a})=154.45\text{J}$$

Since the future positions of the mobile sink node are uncertain, the OSLA schedules the sensor nodes on-line. Therefore, we simulate the mobile sink node's path according to the known Markov parameters to obtain the results for OSLA.

**Figure 8.**
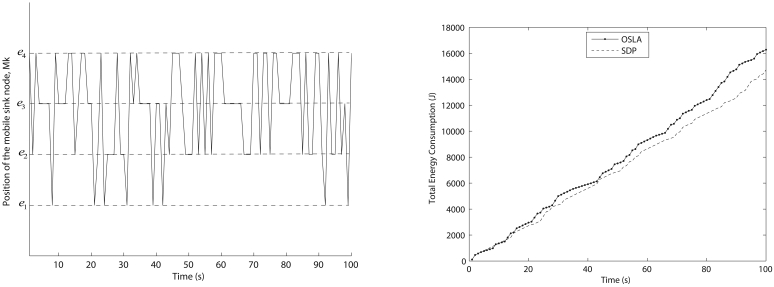
(a) Simulated position of the mobile sink node with time. (b) Total energy consumption obtained by OSLA and SDP for T = 100 s.

The results in Table 3 were averaged over 100 independently simulated paths for the mobile sink node using the known Markov parameters. The mean and standard deviation of the total energy consumption are shown in Table 3. The results in Table 3 obtained from SDP become equal to E{*J*_T_} for very many simulated paths of the mobile sink node. For example, we can see that *J*_100_ = 141.27 with A_1_ whereas $E\left\{J_{\text{T}}^{{\text{A}}_{1}}\right\}=142.54$. Figure 8 shows a simulated path for the mobile sink node using the known Markov parameters and the total energy consumption obtained by OSLA and SDP.

**Figure 9.**
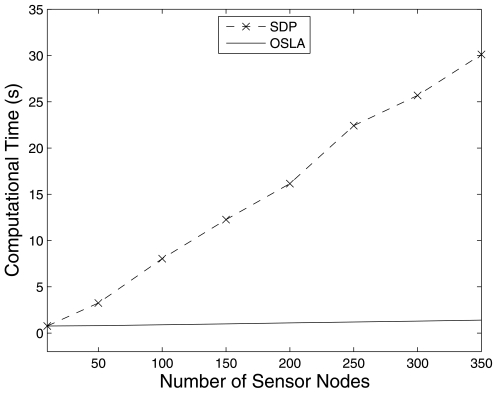
Variation of the computational cost required by the algorithms when the number of sensor nodes increases with 4-State Markov chain.

Since OSLA is a sub-optimal method, on average it consumes more energy than SDP. Even though the results are not promising, the computational cost of the OSLA is very low compared to that of SDP. The number of probable positions (*e_a_*) of the mobile sink node does not affect the computational cost of the OSLA whereas the computational cost of the SDP increases. In order to analyze the effect of the number of sensor nodes on the computational cost of the algorithms, we increase the sensor nodes from 3 to 350 whilst keeping a constant number of probable positions of the mobile sink node. It can be seen from Figure 9 that the computational cost of SDP increases as the number of sensor nodes increases, whereas no significant variation for OSLA was observed.

## 5. Conclusion

In this paper, we studied optimal and sub-optimal methods for scheduling the sensor nodes with a mobile sink node to minimize the total energy consumption for target tracking. An activated sensor node increases its transmission range to maximum only if the measurement could not reach the mobile sink node. Moreover, a sensor node cannot be activated while a previously activated sensor node remains active. We studied the sensor management problem when the path of the mobile sink node is known both fully and partially. We solved the problem optimally using deterministic and stochastic dynamic programming techniques and sub-optimally using rollout and one-step-look-ahead algorithms. The deterministic dynamic programming is best suited for the problem where the path of the mobile sink node is fully known. The limitation of dynamic programming technique is that it cannot be used to solve the problem when the available feasible number of sensor nodes are unknown a priori or varying with time. In such cases, rollout and one-step-look-ahead algorithms are useful. Furthermore, for a big problem, stochastic dynamic programming technique does not solve the problem within a feasible time period due to its high computational cost.

## Figures and Tables

**Table 1.** Properties of the sensor nodes.

Sensor Node (S*_i_*)	Coordinate (L*_i_*)	Transmission Range (*r_i_*)	Active time period (*t_i_*)
S_1_	(150, 150, 0)m	200m	5s
S_2_	(350, 200, 0)m	300m	4s
S_3_	(250, 400, 0)m	200m	2s

**Table 2.** Comparison of cumulative cost *J*_T_ obtained by OSLA, RA-OSLA and DP for different total time horizons.

T (s)	OSLA (J)	RA-OSLA (J)	DP (J)
30	59.0	48.8	40.8
40	72.4	62.4	49.6
50	84.0	74.4	63.2
60	105.6	89.6	76.8
70	123.2	101.6	89.2
80	141.6	118.4	103.2
90	157.2	130.0	114.8
100	175.2	148.8	130.0

**Table 3.** Total average energy consumption obtained by OSLA and SDP with different transition matrices.

T (s)	*J*_T_ with A_1_		*J*_T_ with A_2_	
	OSLA (J)	SDP (J)	OSLA (J)	SDP (J)
30	47.80±5.48	42.50±5.01	51.33±6.26	46.12±5.16
40	64.12±6.53	56.61±5.77	67.86±7.43	61.50±5.92
50	80.24±7.36	70.70±6.41	85.19±8.56	77.10±6.75
60	96.45±7.87	85.14±7.05	101.79±8.80	92.67±7.27
70	112.55±8.89	99.46±7.63	118.97±9.27	107.80±8.04
80	128.34±8.93	113.26±7.98	136.77±9.99	123.38±8.48
90	144.87±9.64	127.84±8.48	153.43±10.91	138.69±9.01
100	160.90±10.15	143.27±9.05	170.82±11.35	155.74±9.65
